# Haploidentical hematopoietic stem cell transplantation using post-transplant cyclophosphamide in patients with inborn errors of immunity: Experience in a reference center in Colombia

**DOI:** 10.7705/biomedica.7560

**Published:** 2024-12-23

**Authors:** Diego Medina, Jhonier Orlando Castro, David Esteban Castro, Estefanía Beltrán, Eliana Manzi, Alexis Antonio Franco, Manuela Olaya

**Affiliations:** 1 Unidad de Trasplante de Progenitores Hematopoyéticos, Servicio de Hemato-Oncología Pediátrica, Departamento Materno Infantil, Fundación Valle del Lili, Cali, Colombia Fundación Valle del Lili Unidad de Trasplante de Progenitores Hematopoyéticos Servicio de Hemato-Oncología Pediátrica, Departamento Materno Infantil Fundación Valle del Lili Cali Colombia; 2 Facultad de Ciencias de la Salud, Universidad ICESI, Cali, Colombia Universidad ICESI Universidad ICESI Cali Colombia; 3 Centro de Investigaciones Clínicas, Fundación Valle del Lili, Cali, Colombia Fundación Valle del Lili Fundación Valle del Lili Cali Colombia; 4 Servicio de Alergología e Inmunología Pediátrica, Departamento Materno-Infantil, Fundación Valle del Lili, Cali, Colombia Fundación Valle del Lili Fundación Valle del Lili Cali Colombia

**Keywords:** Hematopoietic stem cell transplantation, immune reconstitution., trasplante de células progenitoras hematopoyéticas, reconstitución inmunitaria.

## Abstract

**Introduction.:**

Inborn errors of immunity is a diverse group of rare diseases caused by over 400 genetic mutations affecting the immune system and increasing infection susceptibility, autoimmunity, and malignancy. Hematopoietic stem cell transplantation offers a curative option for some inborn errors of immunity, with haploidentical donors providing a viable alternative when identical donors are unavailable.

**Objective.:**

To determine survival, usefulness of weekly chimerism monitoring, immune reconstitution, and complications in patients with inborn errors of immunity who underwent haploidentical hematopoietic stem cell transplantation at a reference center in Colombia.

**Materials and methods.:**

We conducted a retrospective and observational study of a case series of pediatric patients who underwent haploidentical hematopoietic stem cell transplantation with post-transplant cyclophosphamide and follow-up with weekly chimerism. Survival analysis was performed using the Kaplan-Meier method.

**Results.:**

Sixteen patients with haploidentical familial donor transplantation were included. The most frequent diagnosis was severe combined immunodeficiency (n=5). Eleven out of seventeen patients received a non-myeloablative conditioning regimen. Twelve out of sixteen patients developed acute graft-versus-host disease. Out of these, 3 corresponded to grades III-IV. Post-transplant infections affected 14 of the subjects, predominating bacterial agents. Median T-cell chimerism was greater than 80% during the follow-up. Reconstitution of B and T lymphocytes was achieved in more than 80%. Overall survival at five years was 81%. Survival at 100 days was 94%.

**Conclusion.:**

Haploidentical hematopoietic stem cell transplantation using post-transplant cyclophosphamide is a viable alternative for inborn errors of immunity when an identical donor is unavailable. Serial chimerism monitoring is useful for graft follow-up.

## Resumen

**Introducción.:**

Los errores innatos de la inmunidad son enfermedades raras causadas por más de 400 mutaciones genéticas que afectan al sistema inmunológico y aumentan la susceptibilidad a infecciones, la autoinmunidad y las neoplasias malignas. El trasplante de progenitores hematopoyéticos es una opción curativa para algunos errores innatos de la inmunidad y, específicamente, el trasplante haploidéntico es una alternativa viable en ausencia de un donante idéntico.

**Objetivo.:**

Determinar la supervivencia, utilidad del monitoreo semanal del quimerismo, reconstitución inmunológica y complicaciones de los pacientes con errores innatos de la inmunidad sometidos a trasplante haploidéntico de progenitores hematopoyéticos en un centro de referencia en Colombia.

**Materiales y métodos.:**

Se realizó un estudio observacional, retrospectivo, de serie de casos, con pacientes pediátricos sometidos a trasplante haploidéntico de progenitores hematopoyéticos, usando ciclofosfamida después del trasplante y haciendo seguimiento semanal de quimerismo. Se analizó la supervivencia con el método de Kaplan-Meier.

**Resultados.:**

Se incluyeron 16 pacientes con trasplante de donante familiar haploidéntico. El diagnóstico más frecuente fue inmunodeficiencia combinada grave (n = 5). Once de diecisiete recibieron un esquema de acondicionamiento no mieloablativo. La enfermedad aguda de injerto contra huésped se desarrolló en 12 de 16 pacientes y en 3 fue de grado III-IV. Las infecciones posteriores al trasplante afectaron a 14 pacientes, con predominio de agentes bacterianos. La mediana de quimerismo de células T fue mayor al 80 % durante el seguimiento. La reconstitución de linfocitos B y T se logró en más de 11 pacientes La supervivencia global a cinco años fue del 81 % y, a 100 días, fue del 91 %.

**Conclusión.:**

El trasplante haploidéntico de progenitores hematopoyéticos utilizando ciclofosfamida postrasplante, es una alternativa factible en pacientes con errores innatos de la inmunidad en ausencia de un donante idéntico. El monitoreo seriado del quimerismo es útil para el seguimiento del injerto.

Inborn errors of immunity are a heterogeneous group of rare diseases attributed to more than 400 genetic errors affecting different components of the innate and adaptive immune response, resulting in a higher susceptibility to infections, autoimmunity, atopy, dysregulation, and malignancy ^1^. Inborn errors of immunity are classified into ten categories according to the 2022 update of the International Union of Immunological Societies (IUIS), depending on the altered humoral or cellular component ^2^.

In Latin American countries, there is limited information and probably underreporting of inborn errors of immunity. Approximately, between 70 and 90% of people with inborn errors of immunity are not diagnosed ^3^. In Colombia, information is available for less than 15% of the potentially affected population ^4^.

Hematopoietic stem cell transplantation seeks to correct deficits and immune reconstitution and is the only curative procedure for some types of inborn errors of immunity ^5^. An interdisciplinary team decides to transplant a patient and defines the type of donor and conditioning regimen, considering the baseline diagnosis, the patient’s clinical and functional status, the availability of donors, and local resources ^6^.

Currently, the survival of patients with inborn errors of immunity who have undergone hematopoietic stem cell transplantation is between 74 and 90%, depending on the underlying diagnosis ^7^^,^^8^. The effectiveness of this transplantation depends on the immune reconstitution in the host, which means the significant transfer of the cellular and humoral immune response from the donor ^9^.

In general, the donor of choice is a sibling with identical human leukocyte antigen (HLA), as such transplantations have shown the best results. However, because this option is available in only 25 to 30% of cases ^10^, many patients require an alternative donor. This condition limits transplantation opportunities for these patients, especially in countries with scarce resources and low access to unrelated cord blood units or bone marrow donors. Fortunately, a partially compatible (haploidentical) related donor is available in at least 95% of cases, which allows timely treatment ^11^^,^^12^. Haploidentical hematopoietic stem cell transplantation with a post-transplant cyclophosphamide regimen is increasingly used, with satisfactory results ^13^^,^^14^.

Serial evaluation of chimerism is crucial for post-hematopoietic stem cell transplantation follow-up in benign pathologies and neoplasias. This dynamic test allows for the analysis of the evolution of transplanted cells, serving as a predictor of graft failure in inborn errors of immunity ^15^.

This study aimed to determine the survival, immune recovery, and complications of patients with inborn errors of immunity who underwent haploidentical hematopoietic stem cell transplantation at a reference center in Colombia.

## Material and methods

### Type of study

We conducted an observational, descriptive, and retrospective case series study. This study included 17 patients under 19 years old with a diagnosis of inborn errors of immunity who underwent haploidentical hematopoietic stem cell transplantation between January 2012 and December 2020 at a reference center (*Fundación Valle del Lili*) in Cali, Colombia.

The information collected was obtained from medical records. A previous study conducted in three hematopoietic stem cell transplant centers in Colombia contained clinical information on 13 patients ^16^. This study collected new information on serial chimerism and immune reconstitution, for which information is limited in the published literature.

### Definitions

The diagnosis of inborn errors of immunity was based on the 2022 International Union of Immunological Societies (IUIS) classification ^2^. The definitions of severe combined immunodeficiency, chronic granulomatous disease, hemophagocytic lymphohistiocytosis, Wiskott-Aldrich syndrome, leukocyte adhesion deficit, and immunodeficiencies associated with other defects were made according to the criteria established for each disease by the European Society for Immunodeficiencies (ESID) Registry-Working Definitions for Clinical Diagnosis of Inborn Errors of Immunity 2019 ^17^^-^^20^. The conditioning regimen was classified as myeloablative or non- myeloablative ^21^ and was chosen according to the baseline diagnosis ^22^. Post-transplant cyclophosphamide was used to prevent and treat graft- versus-host disease ^23^^,^^24^. Acute and chronic graft-versus-host disease was defined according to established standard criteria ^25^.

Myeloid engraftment was defined as an absolute neutrophil count higher than 0.5 * 10^9^ cells/L for three consecutive days, and platelet engraftment was defined with a count higher than 20 * 10^9^ cells/L without transfusions for three consecutive days ^26^.

Chimerism analysis was performed weekly using short tandem repeats from myeloid engraftment until 100 days after hematopoietic stem cell transplantation and monthly until the first annual visit ^27^. Donor lymphocyte infusion therapy was used in some post-hematopoietic stem cell transplantation patients with unstable mixed chimerism ^28^. CD3+ cells were selected from peripheral blood using immunomagnetic beads to evaluate lineage-specific chimerism. Complete chimerism was defined when more than 95% of donor cells were present, and mixed chimerism when 5 to 95% of donor cells were present.

Primary graft failure was defined as the absence of myeloid engraftment after 28 days post-hematopoietic stem cell transplantation, and secondary graft failure was defined as an absolute neutrophil count inferior to 0.5 * 10^9^ cells/L or donor chimerism less than 5% after the initial graft, no related to drug toxicity or infection ^29^.

Immune reconstitution was assessed in all patients who were alive one-year post-transplantation. It was defined by the minimum threshold of each immune cell type appropriate for age and included patients receiving intravenous immunoglobulin to indicate humoral reconstitution ^30^.

### Statistical analysis

Statistical analysis was performed for all considered variables and selected subgroups. Categorical variables are summarized as proportions, and continuous variables are expressed as medians with their interquartile ranges (IQR). Overall survival analyses were carried out using the Kaplan-Meier method. All analyses were performed with the Stata 14™ statistical software.

### Ethical considerations

This research study was approved by the Comité de ética en investigación biomédica of the Fundación Valle del Lili on January 13, 2021 (approval number 1687). According to the study’s characteristics, the ethics committee has accepted the non-use of informed consent. This work followed the Declaration of Helsinki and the Council for International Organizations of Medical Sciences (CIOMS) guidelines.

## Results

### Demographic and clinical characteristics of the post-transplanted patients

Seventeen haploidentical hematopoietic stem cell transplants were performed in 16 pediatric patients with an inborn error of immunity diagnosis. Of these, one required a new transplantation (table 1).

**Table 1.** Clinical characterization of patients transplanted with haploidentical hematopoietic stem cells

Patient Diagnosis		Age (years)	Gender	BCG-it is pre-HSCT	Relationship to donor	Source	Conditioning	Acute GVHD global classification	Chronic GVHD	Global chimerism (%)	Alive
1	Severe combined immunodeficiency 1 (T-/B-/NK+)	0.5	Female	No	Father	Bone marrow	Flu, Cy, Bu, ATG	II	No	62	Yes
2	Severe combined immunodeficiency 1 (T+/B-/NK- ); Ommen syndrome	1.0	Female	No	Sibling	Bone marrow	Flu, Cy, ATG, TBI = 200 cGy	I	No	30	Yes
3	Severe combined immunodeficiency 1 (T-/B-/NK+)	0.8	Female	Yes	Mother	Peripheral blood	Flu, Cy, ATG, TBI = 200 cGy	II	No	50	No
4	Severe combined immunodeficiency 1 (T-/B+/ NK+)	0.6	Female	Yes	Father	Bone marrow	Flu, Cy, ATG, TBI = 200 cGy	III	No	10	Yes
5	Severe combined immunodeficiency 1 (T-/B-/ NK+)	0.8	Male	No	Father	Bone marrow	Flu, Cy, Mel, ATG	1	No	98	Yes
6	Hemophagocytic lymphohistiocytosis	3.8	Male	No	Father	Bone marrow	Flu, Cy, Bu,ATG,TLI = 750 cGy	-	Yes	60	Yes*
7	Hemophagocytic lymphohistiocytosis	0.6	Female	No	Father	Bone marrow	Flu, Cy, Mel, Thiotepa, ATG	II	No	30	Yes
8	Hemophagocytic lymphohistiocytosis	1.3	Male	No	Sibling	Bone marrow	Flu, Cy, Thiotepa, ATG, TBI = 300 cGy	II	No	100	Yes
9	Chronic granulomatous disease	1.4	Female	Yes	Mother	Peripheral blood	Flu, Cy, Bu,ATG,TLI = 400 cGy	I	Yes	100	Yes*
10	Chronic granulomatous disease	0.5	Male	Yes	Father	Bone marrow	Flu, Cy, Bu, ATG, TLI = 400 cGy	-	No	100	No
11	Wiskott-Aldrich syndrome	2.0	Male	No	Father	Peripheral blood	Flu, Cy, Thiotepa, ATG, TBI = 300 cGy	IV	Yes	10	No
12	Wiskott-Aldrich syndrome	3.2	Male	No	Father	Bone marrow	FLu, Bu, ATG, TBI = 400 cGy	III	No	100	Yes
13	Hyper-IgM syndrome	11.5	Male	No	Sibling	Bone marrow	Flu, Cy, TLI = 750 cGy	-	No	50	Yes
14	Hyper-IgM syndrome	3.2	Male	No	Sibling	Bone marrow	Flu, Cy, Bu, ATG, TLI = 750 cGy	III	Yes	97	Yes*
15	Hyper-IgE syndrome**	19.0	Male	No	Sibling	Peripheral blood	Flu, Cy, Bu, ATG, TBI = 200 cGy	-	No	100	Yes
16	Leukocyte adhesion deficiency type 1	4.4	Male	No	Mother	Peripheral blood	Flu, Cy, Bu, ATG, TLI = 750 cGy	II	No	97	Yes

Pre-HSCT: Pre-hematopoietic stem cell transplantation; BCG-itis: Lymphadenitis caused by the Calmette-Guérin bacillus; GVHD: Graft versus host disease; ATG: Anti-thymocyte globulin; Flu: Fludarabine; Cy: Cyclophosphamide; Bu: Busulphan; Mel: Melphalan, TBI: Total body irradiation, TLI: Total lymphoid irradiation; cGy: centigrays Global chimerism was calculated using the latest chimerism measure of each patient within the first-year post-transplantation.

* Patients receiving immunosuppressive therapy

** Retransplantation

Males predominated (10/16), and the median age at the moment of the transplantation was 1.3 years (IQR = 0.5 - 3.2). The most common diagnosis was severe combined immunodeficiency (5/16), followed by hemophagocytic lymphohistiocytosis (3/16) and chronic granulomatous disease (2/16) (table 2).

**Table 2:** Sociodemographic and clinical characteristics of the patients with inborn errors of immunity who underwent a hematopoietic stem cell transplant between 2012 and 2020

Sociodemographic characteristics (N = 16)		Value
Age (years)		
	Median (IQR)	1.3 (0.5-3.14)
	Range (min-max)	(0.5-19)
Male sex (n)		10
	Type of inborn error of immunity	
	Severe combined immunodeficiencies	5
	Chronic granulomatous disease	2
	Hyper-IgM syndrome	2
	Hemophagocytic lymphohistiocytosis	3
	Wiskott-Aldrich syndrome	2
	Hyper-lgE syndrome	1
	Leukocyte adhesion deficiency type 1	1
Genetic mutations (n)		3
Hematopoietic stem cell transplants (N = 17*)		n
Source of transplant		
	Bone marrow	11
	Peripheral blood	6
Conditioning		
	Myeloablative	6
	Non-myeloablative	11
Anti-thymocyte globulin		15
Donor lymphocyte infusion		
	1 - 5 times	3
Follow-up (months)		
	Median (IQR)	34(14-53)
	Range (min-max)	(1.5 - 78)

IQR: Interquartile range; IEI: Inborn error of immunity; HLH: Hemophagocytic lymphohistiocytosis

* One patient required two transplants.

### Characteristics of the transplant

The source of the hematopoietic stem cells was bone marrow in 11/16 cases. Eleven out of seventeen underwent non-myeloablative conditioning. Three patients required donor lymphocyte infusion therapy using minimum and maximum doses of 0.25 * 10^6^ cells/kg and 10x10^®^ cells/kg, respectively. The median follow-up time was 27.7 months (IQR = 12.7 - 51.3) (table 2).

### Immune reconstitution

The evaluation of immune reconstitution was performed at 12 months. Immune reconstitution of CD3 was observed in 13/14 of the live patients who were evaluated, CD4 reconstitution was found in 11/14, CD8 reconstitution was found in 13/14, NK reconstitution was found in 10/11 since it was not possible to evaluate three patients, B lymphocyte reconstitution was found in 12/14; however, only 5/13 had humoral reconstitution because it was not possible to assess one patient.

### Chimerism

**Figure 1.**
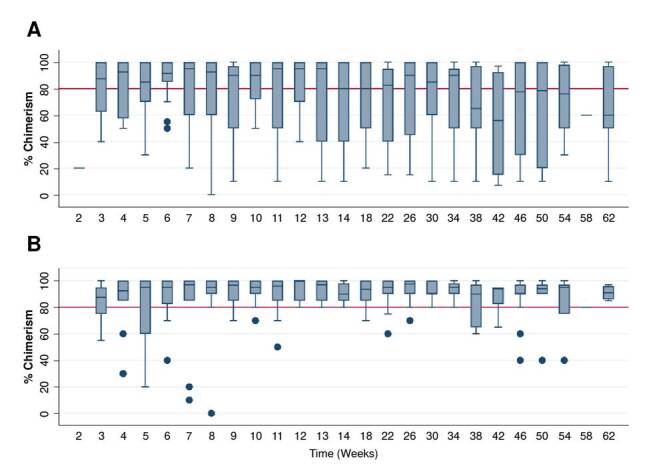
Chimerism progression (donor cell percentage in the transplanted patients) per week: A) Blood total cell chimerism (B) T cells.

A follow-up to determine total chimerism was performed from weeks 2 to 62 post-hematopoletic stem cell transplantation. From weeks 3 to 34, we found a median percentage of donor cells ≥ 80% (figure 1 A). The median T cell chimerism remained above 80% throughout the entire follow-up period, although some extreme values were found in the retransplanted patient (figure 1B).

### Post-hematopoietic stem cell transplantation complications

Acute graft-versus-host disease occurred in 12 transplants; of these, 2 had grade III, and one case had grade IV. Chronic graft-versus-host disease occurred in 24% of patients: in three it was moderate and in one it was severe.

**Figure 2. A):**
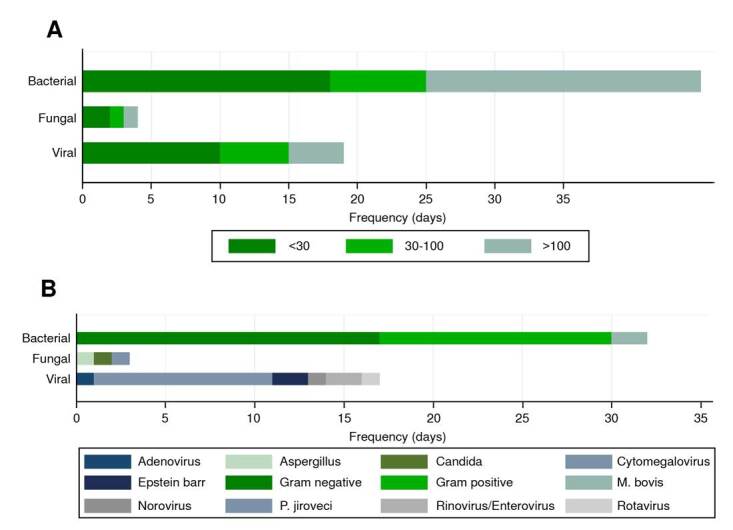
Infectious complications by time of onset (days) post-hematopoietic stem cell transplant. B) Microorganism classification by infection type.

Infectious complications affected 14 transplanted patients, with a total of 52 events: 30 were bacterial, 17 were viral, and 3 were fungal. Most infectious events occurred in the pre-engraftment (n = 17) and late post-engraftment (n = 16). The median times of infection onset after transplantation were 61 days (IQR = 12 - 255) for bacteria, 26 days (IQR = 16-33) for viruses, and 83 days (IQR = 27 - 288) for fungi (table 3). Figure 2A and 2B show the infectious complications according to the onset time and the etiological agent.

**Table 3:** Post-hematopoietic stem cell transplant complications (N = 17)

Complications		Value
Acute graft versus host disease		12
	Grade I-II	9
	Grade III-IV	3
Chronic graft versus host disease		4
	Moderate	3
	Severe	1
Infections		14
Infectious events		52
Type of infection		
	Viral	17
	Bacterial	30
	Mycobacterial	2
	Fungal	3
	Time of infection onset (months)	
	Pre-graft (< 1)	17
	Immediate post-graft (1-3)	19
	Late post-graft (> 3 s)	16
Time of bacterial infection onset (days)		32
	Median (IQR)	60(12-255)
	Range	3-927
Time of viral infection onset (days)		17
	Median (IQR)	26(16-33)
	Range	6-388
Time of fungal infection onset (days)		3
	Median (IQR)	83 (27 - 288)
	Range	27 - 288
Veno-occlusive liver disease		1
Mucositis		
	Moderate-severe	7
Hemorrhagic cystitis		2
Seizure		1
Secondary graft failure		1
Time of death		
	Early (0 - 30 days)	2
	Intermediate (31 -100 days)	0
	Late (> 100 days)	1
Cause of death		
	Aspergillosis	1
	Graft failure	1
	Other (death at home)	1

Pos-HSCT: Post-hematopoietic stem cell transplant; IQR: Interquartile range

Other post-transplant complications were moderate-severe mucositis (n = 7), hemorrhagic cystitis (n = 2), platelet secondary graft failure (n = 1), veno-occlusive liver disease (n = 1), and seizures (n = 1) (table 3).

### Survival

Among the 16 patients in the study, three died, two in post-transplantation early stages (within 30 days) and one later (after 100 days). The causes of death were aspergillosis, graft failure, and unknown in one case because the death occurred at home (table 3).

**Figure 3. A):**
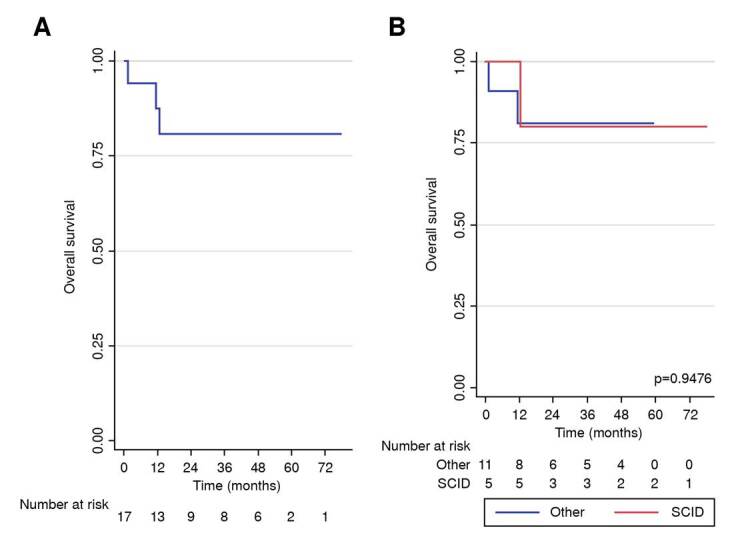
Overall survival of patients with inborn errors of immunity transplanted with haploidentical hematopoietic stem cells; B) Overall survival according to the type of diagnosis (severe combined immunodeficiencies versus others)

Overall survival was 94% at 100 days, 87% at 1 year and 81% at 5 years (figure 3A). Survival at 100 days and at 1 year for those diagnosed with severe combined immunodeficiency was 100%, and at 5 years, it was 80%. Patients with other diagnoses showed survival rates of 91% at 100 days, 81% at one and 5 years (figure 3B).

## Discussion

In this case series, we describe the experience of haploidentical stem cell transplantation in patients with inborn errors of immunity at a reference center in Colombia from 2012 to 2020, with post-transplant cyclophosphamide being the regimen used. Thirteen of the 16 analyzed patients were included in a multicenter study previously conducted in the country ^16^.

Thirteen ouf of sixteen patients showed an overall survival at five years of 81%; this agrees with the survival rates reported in other studies, which vary from 63 to 90% ^31^^,^^32^. In general, patients with severe combined immunodeficiency had a higher survival rate, as published by Gennery *et al*. ^33^. Our patients’ median age at the time of transplantation was higher than that reported in other studies ^34^, which advises performing transplantation during the first six months after birth ^31^.

On the other hand, in patients with diagnoses other than severe combined immunodeficiency, survival rates vary among studies depending on the specific inborn error of immunity ^35^. These results are encouraging in a population in which screening is unavailable and the age at the time of transplantation was higher than six months. Early transplantation ^33^ and screening improve survival ^36^.

The optimal donor is a matched related donor ^34^; however, in most cases, this type of donor is not available, but in our country, the alternative of haploidentical donors is viable and allows access to timely treatment, with survival rates comparable to those of identical transplants ^14^. Contrary to historical reports in which identical transplantation was considered the best option ^26^, the use of unrelated donors for transplantation in cases of inborn errors of immunity donors for hematopoietic stem cell transplantation is now quite common, yielding generally good results. Additionally, haploidentical donors are widely employed in pediatric cases, with excellent outcomes ^37^^,^^38^.

Our results are better than the historical reports in previous studies, such as those by Hladun *et al*. ^31^, who documented significantly higher 10-year survival rates for identical (90%) compared to haploidentical transplants (36%).

Immune reconstitution is one of the main objectives of hematopoietic stem cell transplantation, and the donor can affect the results ^39^. In our cohort, the most common diagnosis was severe combined immunodeficiency, but the T cell reconstitution rate at one year was 93% (13/14 patients). This rate is higher than that reported by Scarselli *et al*. ^40^. The authors showed a T cell reconstitution rate of 76% with severe combined immunodeficiency as the predominant diagnosis and compatible siblings as the most common donor type. It should be noted that the reporting of immune reconstitution is not uniform throughout the studies, which makes it difficult to compare between publications, an aspect to consider in future research.

In our study and others reported, serial chimerism, especially of T lymphocytes, was useful for monitoring the evolution of the graft, allowing the early prediction of transplant rejection and the need for interventions, such as donor lymphocyte infusion or a second transplant in patients with declining chimerism or prolonged mixed chimerism ^28^. In this study, the median T lymphocyte chimerism rate remained above 80% throughout the entire follow-up. This fact is relevant, considering that it is above the minimum level of chimerism required in some pathologies to achieve a cure. The chimerism rate is comparable to the reported in the study of Heimall *et al*. in a cohort of more than 100 patients with severe combined immunodeficiency ^36^, in which the median remained above this value. In our study, follow-up considerations of serial chimerism were useful for determining the need for interventions such as donor lymphocyte infusion therapy or retransplantation in patients who presented a decline in chimerism or prolonged mixed chimerism.

We found an overall acute graft-versus-host disease prevalence of 12/17 transplans, a value within the range of 39.2 and 80%, according to the studies reported by Uppuluri *et al*., Yi *et al*., and Ariffin *et al*. ^32^^,^^35^^,^^41^. The prevalence of acute grades III-IV was 3/12 in our work, similar to that of other published studies ^35^^,^^41^. Novel strategies like the use of anti-thymocyte globulin, low-dose steroids, and abatacept, among others, could decrease the rates of acute and chronic graft-versus-host disease ^42^.

In our study, infections affected 14 out of 16 patients. This rate is similar to that reported by Patiroglu *et al*. ^43^, where100% of their patients developed infections, a finding that highlights the importance of preventing infectious events during the post-transplant period. In our series, bacterial infections predominated, but others have reported viral predominance, such as Patiroglu *et al*. ^43^ and Haddad *et al*. ^44^. The elevated infection rate could be related to the median age at the time of transplantation, which was higher at our institution compared to other studies ^34^. Thakar *et al*. ^45^ mentioned that population-based newborn screening is a valuable strategy for the early identification of infants with inborn errors of immunity, leading to timely hematopoietic stem cell transplantation while preventing infections. This underscores the importance of implementing public health policies to adopt neonatal screening strategies and early referral of patients diagnosed with inborn errors of immunity in our country and throughout Latin America.

Some limitations of this study include its retrospective design and the collection of information from electronic medical records, which may be associated with incomplete data. In addition, the findings do not reflect the country’s context, as the study was conducted at a single center.

Haploidentical transplant and post-transplant cyclophosphamide in patients with immunodeficiencies facilitated patients’ access to hematopoietic stem cell transplantation, with survival rates like those reported using other donor types. T-cell chimerism is useful during the follow-up to identify engraftment and decide the need for interventions. A screening test for inborn errors of immunity would allow earlier treatment and further improved survival rates in our circumstances.
